# Gender differences in the evaluation of care for patients with type 2 diabetes: a cross-sectional study (ZODIAC-52)

**DOI:** 10.1186/s12913-018-3086-x

**Published:** 2018-04-10

**Authors:** Steven H. Hendriks, Marco H. Blanker, Yvonne Roelofsen, Kornelis J. J. van Hateren, Klaas H. Groenier, Henk J. G. Bilo, Nanne Kleefstra

**Affiliations:** 1 Diabetes Centre, Isala, Zwolle, the Netherlands; 2 Department of General Practice, University of Groningen and University Medical Center Groningen, Groningen, the Netherlands; 3 Medical Research Group, Langerhans, Ommen, the Netherlands; 4 Department of Internal Medicine, University of Groningen and University Medical Center Groningen, Groningen, the Netherlands; 5 Department of Internal Medicine, Isala, Zwolle, the Netherlands

**Keywords:** Type 2 diabetes mellitus, Evaluation of care, Primary health care, Health care surveys

## Abstract

**Background:**

Little is known about the association between patient-related factors and patients’ evaluation of care.

Aim was to investigate which patient-related factors are associated with patients’ evaluation of care in men and women with type 2 diabetes (T2D) in primary care.

**Methods:**

This cross-sectional study included 1102 patients with T2D from 52 general practices. We measured patients’ evaluation with the EUROPEP questionnaire and collected demographic, clinical and psychological data from questionnaires and health records. Stepwise linear regression analyses were used.

**Results:**

The location where the questionnaire was completed (at home or at the general practice) was associated with all outcomes in men and women. Next to this, in men, explanatory factors for the care provider EUROPEP subscale were use of insulin, having some problems with T2D self-care and coffee consumption (R^2^ 8.4%); coffee consumption was associated with the general practice subscale (R^2^ 4.0%). In women, well-being, quality of life, following a general diet, and use of oral glucose-lowering drugs were associated with the care provider subscale (R^2^ 16.8%). For the general practice subscale, well-being and age were explanatory factors (R^2^ 9.4%).

**Conclusions:**

Only a few factors were found to be associated with patients’ evaluation of care for men and women with T2D. Taken together, these factors explained only a small part of the variance of the EUROPEP scores. This explained variance was largely attributable to the location where the questionnaire was completed. We therefore advise to be aware of the possible consequences of filing-out questionnaires about patients’ evaluation of care at the general practice.

**Trial registration:**

NCT01570140 (Clinicaltrials.gov). Registered 29 March 2012.

## Background

Patients’ evaluation of delivered care is becoming an increasingly important quality outcome of health care [1]. In some countries, this evaluation already is a regular part of the evaluation of care for patients with chronic diseases.

Variation in patient evaluations could reflect differences in the way general practitioners (GPs) deliver care but could also reflect differences between patients [2]. Higher age, having a chronic disease, having a higher risk for cardiovascular diseases and a higher frequency of attendance are all patient-related factors associated with a more positive evaluation of care [2, 3]. Self-rated health shows conflicting results in relation to patients’ evaluation of care [3, 4]. In patients with type 2 diabetes mellitus (T2D), a higher HbA1c and receiving insulin therapy are described to be associated with a more positive evaluation [5, 6]. Knowledge of the extent to which patient characteristics are associated with variation in patient evaluations of care allows accounting for these differences when comparing practice populations and GPs [2]. Furthermore, insight into these factors may lead to a more positive patient experience.

Little is known about the extent to which patient-related factors are associated with patients’ evaluation of care. One study, which was conducted amongst patients with osteoarthritis, described that 27% of the variance in patient evaluation could be explained by patient-related factors [7]. The degree to which patient-related factors contributes to the prediction of evaluation of care in patients with T2D is unknown. Also, it is unknown whether there are gender differences. Sex and gender research in the field of T2D showed that the negative impact of T2D on different healthcare outcomes might be higher among women compared to men [8]. This might also result in gender differences in evaluation of care. Women may base their judgment of delivered care on other factors compared to men, as in women emotional factors appear to influence the decision process more than is the case in to men [9]. The degree to which patient-related factors are associated with patients’ evaluation of care could therefore be different between men and women. Identifying possible differences in patient-related factors between men and women may call for development of more gender-specific care for patients with T2D. The aim of the current study was to investigate which patient-related demographic, psychological and clinical factors are associated with patients’ evaluation of delivered care in T2D patients, with a focus on gender differences.

## Methods

### Study population and design

The current study was performed using baseline data from an observational prospective cohort study. The design and details of this study have been published previously [10]. Briefly, this study was initiated with the primary aim to investigate the influence of the use of an online care platform on the health-related quality of life (HRQoL) of patients with T2D treated in primary care. All patients with T2D in 52 general practices in the Drenthe region (the Netherlands) were asked by their practice nurses to fill out questionnaires including questions on HRQoL, well-being and degree of self-reliance. Additionally, all patients were given access to a care platform on the internet, which provided laboratory results based on the yearly check-ups, educational modules and a module to start a self-chosen process of lifestyle intervention through the platform. The use of this platform was entirely voluntary.

Questionnaires were filled out at the general practice on a tablet computer. Many patients experienced problems with this method during the first half of the inclusion period. Therefore, during the second half of the study period, patient could also fill out the questionnaires at home on paper.

**Fig. 1:**
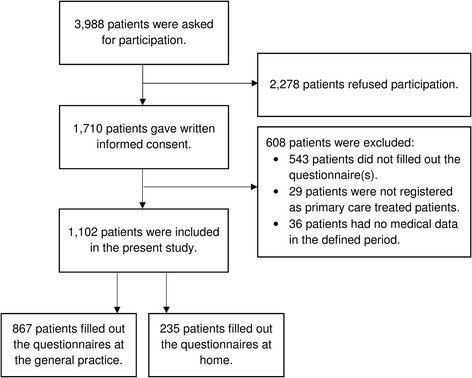
Flowchart of patient inclusion

Patients were included from May 2012 till September 2014. A total of 1710 (42.9%) out of 3988 patients, who were asked to participate, gave written informed consent. The final study sample consisted of 1102 (64.4%) patients; see for more details the flowchart in Fig. 1.

### Patients’ evaluation of care questionnaire

The European Task Force on Patient Evaluations of General Practice (EUROPEP) questionnaire was used to assess patients’ evaluation of care [3, 11]. This internationally validated questionnaire contains 23 items, which measure different aspects of care. The patients respond to each item on a five-point Likert scale ranging from ‘poor’ to ‘excellent’ or by choosing for the category ‘not applicable’. The EUROPEP questionnaire covers two dimensions of care: a care provider evaluation (items 1–17) and a general practice evaluation (items 18–23) [3]. In the current study, the mean scores of both dimensions were used. This questionnaire was filled out at baseline.

### Survey data

Three months later, a range of validated questionnaires which measure the perceived quality of life (EQ-5D) (EQ-VAS) [12, 13], well-being (WHO-5) [14], diabetes-related distress (PAID-5) [15] and self-reliance (SDSCA) [16] were filled out. For the EQ-5D, WHO-5 and the PAID-5 questionnaire, we calculated a sum score. For the SDSCA questionnaire the general diet, exercise and foot-care subscales scores were calculated and the individual items concerning full-fat dairy products and fruit and vegetables were used. Additionally, we collected information on daily occupation, education level, family history of cardiovascular diseases (CVD), smoking, alcohol consumption, coffee consumption, tea consumption, problems with diabetic self-care, concerns about hypoglycemia, attending psychological care and fall accidents.

We categorized daily occupation into having a job (full-time or part-time), being unemployed or incapacitated, being retired or being a housewife or –man; educational status as low, intermediate or high; problems with diabetic self-care into: no, a little, some, or huge problems; and concerns about hypoglycemia as: no, a little or huge concerns. For coffee and tea consumption we used a continuous scale. Smoking, alcohol consumption, attending psychological care and fall accidents were handled as dichotomous (yes/no) variables. We categorized the location where the questionnaire was completed into two groups; at the general practice or at home.

### Health record data

We obtained clinical data and additional demographic data from the personal health record systems of the GPs. These data were collected during the annual check-up of the patients and were already routinely sent to our Diabetes Centre for benchmark and study purposes. Clinical data obtained in the period from 9 months before and 5 months after the EUROPEP questionnaire were used in this study. After informed consent of included patients, we combined these clinical data with the results of the collected questionnaires to assemble an anonymized dataset.

The following data were used in the current study: age, gender, duration of diabetes, BMI, HbA1c, systolic and diastolic blood pressure, total cholesterol, LDL-cholesterol, HDL-cholesterol, serum creatinine, the presence of microvascular complications, the presence of macrovascular complications and the use of glucose lowering, the use of lipid lowering and the use of antihypertensive medication. We defined the presence of microvascular complications as having diabetic retinopathy, albuminuria and/or diabetic peripheral neuropathy, and the presence of macrovascular complications as (a history of) angina pectoris, myocardial infarction, percutaneous transluminal coronary angioplasty, coronary artery bypass grafting, stroke or transient ischemic attack or the use of thrombocyte aggregation inhibitors. Glucose lowering therapy was categorized into: diet, oral blood glucose lowering therapy and insulin therapy (with or without oral therapy).

### Statistical analysis

Statistical analyses were performed using SPSS version 20 (IBM Corporation, Somers, NY, USA). We used multiple imputation for missing data on the independent variables, assuming that data was missing at random (MAR) or completely at random (MCAR). Ten imputated datasets were created. Baseline results are expressed as mean with standard deviation (SD) or median with interquartile range [IQR] for normally distributed and non-normally distributed data, respectively. Categorical variables are described in numbers and percentages. Differences were considered to be significant at a *p*-value of < 0.05. A prediction model was built to find explanatory variables for the EUROPEP outcomes. For this, we entered all available parameters in stepwise linear regression models with bidirectional elimination (PIN = 0.05 and POUT = 0.1) with the mean score of the two EUROPEP subscales as the dependent variables. We performed the analyses for men and women separately. Final models are presented. The degree to which the models determined EUROPEP subscale scores was evaluated by the explained variance, shown as adjusted R^2^. Before analyses, the presence of multicollinearity was tested between the WHO-5 and EQ-5D scores.

## Results

### Patient characteristics

Baseline results of the study population are described in Table 1. Fifty-six percent of the patients were male. Mean age was 65.5 (SD 9.5) years in men and 63.9 (10.5) years in women. Men had a higher score on the WHO-5 questionnaire and had a higher education level compared to women. Men also had more often micro- and macrovascular complications and used alcohol more frequently compared to women. Women had contact with psychological caregivers more frequently compared to men and the percentage of housewives was much higher than the percentage of housemen.

**Table 1:** Baseline variables for men and women with type 2 diabetes

Variables	Men	Women	*p*-value
N	616 (56%)	486 (44%)	
Mean age	65.5 (±9.5)	63.9 (±10.5)	0.012
EUROPEP			
Median care provider score	4.4 (4.0 – 4.9)	4.5 (4.0 – 4.9)	0.063
Median general practice score	4.2 (3.8 – 4.6)	4.2 (3.8 – 4.7)	0.832
Median WHO-5 sum score	76 (68 – 84)	72 (60 – 80)	< 0.001
Median EQ-5D sum score	0.9 (0.8 – 1.0)	0.84 (0.78 – 1.00)	< 0.001
Median EQ-VAS score	80 (70 – 90)	80 (61 – 88)	0.007
Median PAID-5 score	5 (0 – 15)	5 (0 – 20)	0.016
SDSCA items (median scores)			
General diet	6 (5 – 7)	6 (5 – 7)	0.171
Fruit and vegetables	6 (5 – 7)	6 (5 – 7)	< 0.001
Less Fat	5 (4 – 6)	6 (5 – 6)	< 0.001
Exercise	4 (2.5 – 6.0)	4 (2.5 – 5.5)	0.728
Foot-care	1 (0 – 3.5)	1.5 (0 – 3.5)	0.060
Level of education			
Low	179 (29.1%)	197 (40.5%)	< 0.001
Mediate	264 (42.9%)	220 (45.3%)	–
High	173 (28.1%)	69 (14.2%)	–
Occupation			
Job	176 (28.6%)	116 (23.9%)	< 0.001
Retired	368 (59.7%)	206 (42.4%)	–
Unemployed/ incapacitated	61 (9.9%)	38 (7.8%)	–
Housewife/−man	11 (1.8%)	126 (25.9%)	–
Problems with DM self-care			
No	390 (63.3%)	313 (64.4%)	0.683
A little	160 (26.0%)	131 (27.0%)	–
Some	45 (7.3%)	26 (5.3%)	–
Huge	21 (3.4%)	16 (3.3%)	–
Fall accidents	149 (24.2%)	144 (29.6%)	0.041
Vascular diseases in family	270 (43.8%)	249 (51.2%)	0.019
Contact with psychological caregivers	26 (4.2%)	41 (8.4%)	0.004
Worries about hypoglycemia			
No	457 (74.2%)	328 (67.5%)	0.052
A little	98 (15.9%)	91 (18.7%)	–
Huge	61 (9.9%)	67 (13.8%)	–
Smoking	110 (17.9%)	97 (20.0%)	0.371
Alcohol usage	409 (66.4%)	179 (36.8%)	< 0.001
Median coffee usage	4 (3 – 6)	3 (2 – 4)	< 0.001
Median tea usage	2 (0 – 3)	2 (1 – 4)	< 0.001
Median BMI (kg/m2)	28.7 (26.2 – 31.5)	29.6 (26.8 – 33.5)	< 0.001
Median diabetes duration (years)	6.8 (3.2 – 9.8)	6.8 (3.1 – 10.5)	0.436
Median HbA1c (mmol/mol)	49 (44 – 54)	48 (44 – 53)	0.605
Median systolic blood pressure (mmHg)	136 (128 – 144)	132 (124 – 142)	0.009
Median diastolic blood pressure (mmHg)	80 (70 – 84)	78 (70 – 82)	0.087
Median cholesterol (mmol/L)	4.1 (3.6 – 4.8)	4.5 (3.9 – 5.1)	< 0.001
Median HDL (mmol/L)	1.1 (1.0 – 1.3)	1.4 (1.2 – 1.6)	< 0.001
Median LDL (mmol/L)	2.3 (1.8 – 2.8)	2.4 (1.9 – 3.0)	0.003
Median creatinine (μmol/L)	85 (75 – 96)	68 (61 – 78)	< 0.001
Microvascular complications	245 (39.8%)	122 (25.1%)	< 0.001
Macrovascular complications	226 (36.7%)	107 (22.0%)	< 0.001
Diet	105 (17.0%)	101 (20.8%)	0.114
Oral medication	426 (69.2%)	313 (64.4%)	0.096
Insulin use	85 (13.8%)	72 (14.8%)	0.632
Use of antihypertensive drugs	462 (75.0%)	361 (74.3%)	0.785
Use of lipid lowering drugs	499 (81.0%)	361 (74.3%)	0.007

Values are depicted as number (%), means (± SD), or median (IQR)

*Abbreviations*: *BMI* body mass index, *HDL* high density lipoprotein, *LDL* low density lipoprotein

The median EUROPEP score for the evaluation of the care provider was 4.4 (IQR 4.0 – 4.9) in men and 4.5 (4.0 – 4.9) in women. The median EUROPEP score for the evaluation of the general practice was 4.2 (3.8 – 4.6) in men and 4.2 (3.8 – 4.7) in women.

### Variables associated with patient’s evaluation of the care provider

In multivariable analyses for men, the location where the questionnaire was completed, the use of insulin, having some problems with DM self-care and coffee consumption were associated with the care provider subscale of the EUROPEP (Table 2). The explained variance of this multivariate linear model was 8.4%.

**Table 2:** Factors associated with the EUROPEP score in men and women with type 2 diabetes

	Men (616)			Women (486)		
Care provider evaluation^b^	Adjusted R^2^ total model (%) = 8.4			Adjusted R^2^ total model (%) = 16.8		
	B (95%BI)	*p*-value	R^2a^	B (95%BI)	*p*-value	R^2a^
Location of completing questionnaire^d^	− 0.330 (− 0.425, − 0.235)	< 0.001	6.6	− 0.448 (− 0.560, − 0.336)	< 0.001	10.9
Use of insulin	0.135 (0.020, 0.250)	0.021	0.6	ns		
Some problems with DM self-care	− 0.138 (− 0.338, − 0.029)	0.020	0.6	ns		
Coffee consumption	−0.018 (− 0.033, − 0.003)	0.022	0.6	ns		
Well-being (WHO-5 sum score)	ns			0.007 (0.004, 0.010)	< 0.001	3.3
Quality of life (EQ-5D sum score)	ns			−0.340 (−0.630, − 0.050)	0.022	0.8
General diet	ns			0.034 (0.008, 0.061)	0.012	1.3
Use of oral glucose lowering drugs	ns			0.098 (0.004, 0.192)	0.041	0.5
General practice evaluation^c^	Adjusted R^2^ total model (%) = 4.0			Adjusted R^2^total model (%) = 9.4		
	B (95%BI)	*p*-value	R^2a^	B (95%BI)	*p*-value	R^2a^
Location of completing questionnaire^d^	− 0.246 (− 0.360, − 0.132)	< 0.001	2.6	− 0.329 (− 0.463, − 0.195)	< 0.001	3.9
Coffee consumption	−0.030 (− 0.048, − 0.012)	0.001	1.4	ns		
Well-being (WHO-5 sum score)	ns			0.007 (0.004, 0.010)	< 0.001	4.1
Age	ns			0.007 (0.002, 0.013)	0.004	1.4

^a^Contribution to the adjusted R^2^ per variable

^b^Care provider subscale of the EUROPEP questionnaire

^c^General practice subscale of the EUROPEP questionnaire

^d^Filled-out at home compared to filled-out at the general practice

In women, the location where the questionnaire was completed, well-being, quality of life, following a general diet, and the use of oral glucose lowering drugs were associated with the EUROPEP care provider outcome (R^2^ 16.8%).

### Variables associated with patient’s evaluation of the general practice

In men, associations with the general practice subscale of the EUROPEP were found for the location where the questionnaire was completed and for coffee consumption (Table 2). The explained variance of this multivariate linear model was (R^2^ 4.0%). In women, the location where the questionnaire was completed, well-being and age were associated with the evaluation of the general practice (R^2^ 9.4%).

### Location where the questionnaires were completed

**Fig. 2:**
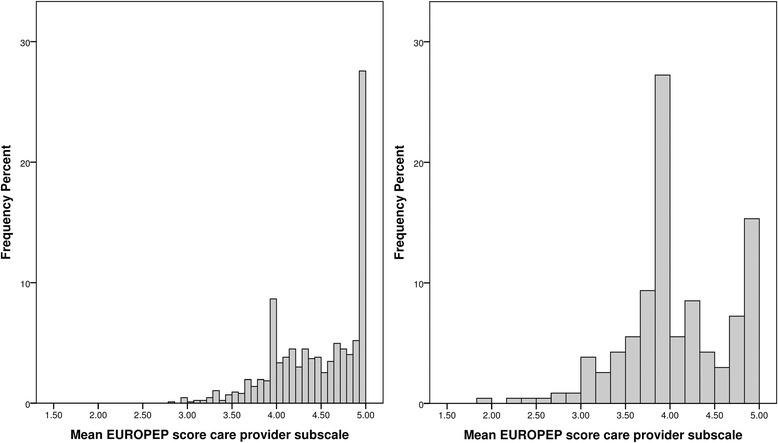
Distribution of EUROPEP scores for the care provider subscale for patients who have filled out the questionnaire at the general practice (left graph) or at home (right graph)

**Fig. 3:**
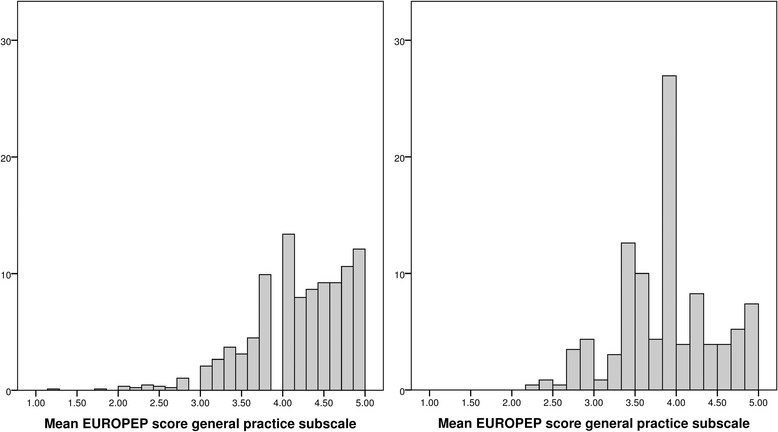
Distribution of EUROPEP scores for the general practice subscale for patients who have filled out the questionnaire at the general practice (left graph) or at home (right graph)

We performed post-hoc analyses on the location where the questionnaires were completed. Figures 2 and 3 shows the distribution the EUROPEP mean scores for both subscales stratified to the location of completing the questionnaires. In all graphs, many high scores were observed in patients who filled out the questionnaire at the general practice.

In men who filled out the questionnaire at the general practice, the median EUROPEP scores for the care provider and general practice subgroups were 4.5 (4.1 – 4.9) and 4.3 (3.8 – 4.7), respectively. In men who filled out the questionnaires at home, the median EUROPEP scores for the care provider and general practice subgroups were 4.0 (3.8 – 4.6) and 4.0 (3.36 – 4.3), respectively.

In women who filled out the questionnaire at the general practice, the median EUROPEP scores for the care provider and general practice subgroups were 4.6 (4.1 – 5.0) and 4.3 (3.8 – 4.7) respectively. In women who filled out the questionnaires at home, the median EUROPEP scores for the care provider and general practice subgroups were 4.0 (3.8 – 4.6) and 4.0 (3.5 – 4.2), respectively.

## Discussion

In men, filling out the EUROPEP questionnaire at the general practice, using insulin, not having some problems with diabetes self-care and less coffee consumption were associated with a better patient evaluation of care, as measured with the EUROPEP questionnaire. In women, filling out the EUROPEP questionnaire at the general practice, a higher degree of well-being, a lower quality of life, following a general diet, using oral glucose lowering drugs and a higher age were associated with higher EUROPEP scores. However, the explained variance of these factors together was low in both genders. Besides this, the location where questionnaires were completed was a predominant factor in all analyses.

In women, we found an association between a higher degree of well-being and higher EUROPEP scores on both subscales. This association was also found by Roseman et al. in patients with osteoarthritis [7]. In our study, an association between a lower health-related quality of life and a higher EUROPEP score on the care provider subscale was also observed in women. No associations between well-being, health-related quality of life and EUROPEP scores were found in men. This may indicate that the evaluation of care is more influenced by personal feelings in women compared to men. The association between higher age and higher EUROPEP scores, as found in other studies [2, 3, 7], was confirmed only for women in our study. The finding that the use of insulin is associated with a higher EUROPEP score on the care provider subscale is not in line with previous literature [5]. Other associations that have not been described before are that less coffee consumption is associated with higher EUROPEP scores in men and that following a diet or using oral glucose lowering drugs are associated with higher EUROPEP score on the care provider subscale in women.

Men and women, who have filled out the questionnaires at home, had approximately a 0.5 point lower median score on the care provider subscale and a 0.3 point lower median score on the general practice subscale compared to men and women who have filled out the questionnaires at the general practice. As almost all mean EUROPEP subscale scores ranged between 3 and 5, a difference of 0.5 is relevant. At the general practice, the questionnaires were filled out on a tablet computer. Some patients experienced practical problems with the use of these tablets and therefore they needed help. The presence of a care provider or assistant in the same room may have led to giving desired answers, also called the ‘yes’ saying bias. This is a culturally based tendency to agree with others, which is mostly seen in face-to-face interviews [17]. The questionnaires filled out at home were directly sent to our Diabetes Centre without intervention of the care provider. These patients were possibly more honest about the received care as they knew that the care provider could not observe the answers. Furthermore, these patients probably did not experience time pressure, giving the respondents more time to think, which may have led to other responses [17]. It should be mentioned that it was not our aim to investigate the influence of the location where the questionnaire was completed in particular. Though, significant lower EUROPEP scores were found in the total population in the period when it was possible to fill out the questionnaire at home (data not shown). This finding is strongly suggestive for an influence of the location where the questionnaire was filled out, which could be an objective in future research.

One may conclude that patients’ evaluation of care is not much depending on patient-related factors, because only a few patient-related factors were associated with the EUROPEP scores and the explained variance of these factors together was low. However, some important patient-related factors were not taken into account. It may be that patients’ evaluation of care is depending on the ability of patients to navigate through the healthcare system, their perceived self-efficacy and their motivation to play an active role in the care process. These aspects were not investigated in the present study. It may also be that the evaluation of care is more depending on the quality of the general practice and the behavior and character of the care provider. In previous studies, a lower GP’s age, a comparatively low number of listed patients per GP, working in a single-handed practice and performing clinical audits were associated with a more positive evaluation of care [4, 18]. Associations with these factors are likely to be especially present in men, as the explained variance in men of all variables, excluding the location where the questionnaire was completed, was less than 2%. The low explained variance could also be the result of a ceiling effect. This has led to a restriction of range in EUROPEP scores. In such a homogeneous group it is hard to find predictive factors and there is not much variance that could be explained. It is certainly possible that this ceiling effect reflects reality, since patients treated in primary care in the Netherlands are quite satisfied with the delivered care [19].

It should be noticed that this study is a cross-sectional study and that no conclusions can be drawn about causality. Furthermore, due to the explorative character of our study all associations found could be a matter of coincidence and should therefore be tested in further studies. Lastly, the questionnaires were derived from a study with as primary aim to investigate the effect of e-Health on quality of life. Selection bias has occurred in this study as participants were more often men, younger and had a shorter duration of diabetes compared to non-participants [20]. However, it is unclear whether this selection bias has influenced the results in the current study.

## Conclusions

Only a few patient-related factors were found to be associated with T2D patients’ evaluation of primary care and these factors together explained only a small part of the variance of the EUROPEP scores, especially in men. This explained variance was largely attributable to the location where the questionnaire was completed. We therefore advise to be aware of the possible consequences of filing-out questionnaires about patients’ evaluation of care at the general practice. It should be investigated in future research whether a causal influence of the location where the questionnaire was completed on the evaluation of care exists. Furthermore, gender differences in the association between other patient-related factors and patients’ evaluation of care, such as the motivation to play an active role in the care process and perceived self-efficacy should be further investigated.

## Abbreviations

CVD: Cardiovascular diseases (CVD)
EUROPEP: European task force on patient evaluations of general practice
GPs: General practitioners
HRQoL: Health-related quality of life
SD: Standard deviation
T2D: Type 2 diabetes
